# Investigating the Analytical Variability and Agreement of Manual Leukocyte Quantification Methods in Eastern Box Turtles (*Terrapene carolina carolina*)

**DOI:** 10.3389/fvets.2019.00398

**Published:** 2019-11-12

**Authors:** John M. Winter, Nicole I. Stacy, Laura A. Adamovicz, Matthew C. Allender

**Affiliations:** ^1^Wildlife Epidemiology Laboratory, College of Veterinary Medicine, University of Illinois, Urbana, IL, United States; ^2^Department of Diagnostic, Comparative, and Population Medicine, College of Veterinary Medicine, University of Florida, Gainesville, FL, United States

**Keywords:** hemacytometer, hematology, manual methodology, reptile, white blood cell count, white blood cell estimate

## Abstract

Leukogram evaluation provides valuable information about inflammation, infection, and stress in free-living and zoo-maintained wildlife. While multiple protocols for quantifying leukocytes are available in reptiles, agreement between methods is infrequently described and analytical variability (including repeatability and reproducibility) has not been critically evaluated. This study addresses these knowledge gaps for two hematological methods in eastern box turtles (*Terrapene carolina carolina*): Avian Leukopet^™^ (LO) and total white blood cell (WBC) estimates from blood films (EST). The objectives of this study were (1) to evaluate agreement in total WBC and individual leukocyte counts between the LO and EST methods, (2) to document repeatability (intra-assay variability) and reproducibility (inter-assay variability) for the LO method, and (3) to investigate whether biological drivers of WBC counts differ between quantification methods. Box turtles (*n* = 120) were sampled from five study sites in Illinois during the 2018 active season. The LO method produced significantly higher WBC counts than the EST method, and constant and proportional error was variable for each leukocyte type. The LO method demonstrated an intra-assay variability of 8.2% and an inter-assay variability of 12%, independent of biological variation. WBC counts were significantly affected by age class using both LO and EST methods, but WBC differences between locations and sexes were only observed using the LO method. These findings emphasize the importance of considering leukocyte determination method when analyzing reptilian hematology results. The inherent variability in currently available methods creates uncertainty in resulting data and highlights the need of a gold standard for reptilian WBC quantification.

## Introduction

Monitoring health is critical to the conservation of any wild species, as the viability of a population is inseparable from its health status (1). However, baseline health data including clinical pathology values (e.g., hematology, plasma biochemistries, protein electrophoresis), pathogen prevalence, and contaminant concentrations are infrequently available for free-living animals. This lack of information can complicate the design and interpretation of comprehensive health studies. Establishing baseline clinical pathology data facilitates monitoring for trends in overall health status and can inform more effective conservation management strategies (2). Hematology is one of the most commonly-used veterinary health assessment tools due to ease of performance, cost-effectiveness, and wide availability in diagnostic and research settings.

Leukogram changes can indicate inflammation, infection, and stress associated with poor health or unsuitable environmental conditions, highlighting the utility of hematologic indices for health assessment in sentinel species like reptiles (3, 4). While mammalian hematology can be performed using automated cell counters, reptilian hematologic analyses require manual methods due to their characteristic nucleated erythrocytes and thrombocytes. Manual leukocyte counting methods used in reptiles include total white blood cell (WBC) estimates from blood films (EST), Natt and Herrick's (NH) direct counts, and indirect leukocyte quantification using the Avian Leukopet kit (LO). The proper application of manual hematology methods is technically challenging and produces more imprecise leukocyte estimates than automated analyzers in mammals (5). However, the analytical variability of these methods has not yet been assessed in reptiles; limiting our understanding of the random error inherent in these approaches.

Similarly, hematologic method comparison studies are limited in reptiles. In Galápagos tortoises (*Chelonoidis* spp.), significant differences in leukocyte quantification were reported between the NH and LO quantification methods and the NH method exhibited better agreement with the EST method (6). Discrepancies in leukocyte counts between different methods have also been documented in loggerhead sea turtles (*Caretta caretta*) (7, 8) and leatherback sea turtles (*Dermochelys coriacea*) (9). However, the degree to which different hematologic methods agree has not yet been determined in terrestrial turtles.

Conclusions drawn from studies investigating hematologic variation due to temporal, spatial, and demographic factors assume that measurements accurately reflect the true value of the analyte. Thus, understanding method-based variability in leukocyte quantification is critical for appropriately interpreting wildlife health data and comparing health status between populations and studies. Characterizing the analytical variability of hematologic assays may encourage researchers to use the most appropriate leukocyte quantification methods, reduce the occurrence of erroneous findings, and consequently allow for more accurate conclusions to be drawn leading to improved wildlife health research.

This study addressed the following specific objectives using blood samples from free-living eastern box turtles (*Terrapene carolina carolina*): (1) Determine level of agreement between the EST and the LO methods, (2) Determine repeatability (intra-assay variability) and reproducibility (inter-assay variability) of the LO method, and (3) Assess whether biological drivers of leukocyte counts are similar between hematologic methods.

## Materials and Methods

### Field Techniques

Eastern box turtles (EBT) were collected from five field sites in Illinois (Collison, Forest Glen, Kennekuk, Kickapoo, and Forbes) using dog-assisted capture as previously described (10). Turtles were weighed, assigned to an age class (adults were >200 g, juveniles were <200 g), and sexed using a combination of sexually dimorphic traits (11). Complete physical examinations were conducted by a single observer (LA). Turtles with clinical signs of illness (ocular/nasal discharge, oral plaques, open-mouth breathing, etc.) or active injuries were categorized as “unhealthy,” while turtles with no clinical signs of illness or fully-healed injuries were considered “apparently healthy.”

Blood (<0.08% body weight) was collected via the subcarapacial sinus, immediately placed into lithium heparin microtainers (Becton, Dickinson and Company, 1 Becton Drive, Franklin Lakes, NJ), and stored on ice packs until processing (2–6 h after collection). Blood that was obviously lymph contaminated or clotted was not used in analyses. All activities involving live animals were approved by the University of Illinois Institutional Animal Care and Use Committee (Protocol 18000).

### Complete Blood Counts

Blood smears were made using heparinized samples immediately upon arrival at the laboratory. Slides were air-dried, then stained using a modified Wright-Giemsa method (Hema 3^™^ Stat Pack, Fisher Scientific, 300 Industry Drive, Pittsburgh, PA). Total leukocyte quantification was then performed using two methods: (1) the Avian Leukopet^™^ Kit (Vetlab Supply, Palmetto Bay, FL) with 100-WBC differential counts from corresponding blood films, and (2) WBC estimates and 100-WBC differential counts from blood films. The LO procedure was performed per manufacturer's instructions and read on a Bright-line hemacytometer (Hausser Scientific, Horsham, PA, USA). Briefly, blood samples were repeatedly inverted to ensure a homogeneous blood cell mixture, 25 μL of heparinized whole blood was gently mixed with 750 μL of phloxine stain, the mixture was incubated for 10 min, equal volume aliquots were loaded into each hemacytometer chamber, and the hemacytometer was left undisturbed for 10 min prior to cell counting. Stained heterophils and eosinophils were counted in all nine squares of the hemacytometer grids, and total WBC count was determined using the following equation (12):

$$\begin{array}{l} \left[\frac{Hemacytometer\;side\;1+Hemacytometer\;2}{\begin{array}{c} \%Heterophils+\%Eosinophils\;\\ \left(from\;differential\;count\right) \end{array}}\;\times 1.1\times 16\times 100\right] \end{array}$$

Total white blood cell estimates (EST) were performed by a single observer (JW) at 40x magnification by counting all leukocytes across 10 different fields of the monolayer. The results were averaged and multiplied by 2000 to calculate the number of white blood cells per microliter (13). One-hundred WBC differentials were performed under oil immersion by a single observer (LA).

#### Intra-Assay Variability of the Avian Leukopet^™^

Three hemacytometers were loaded and reviewed from a single phloxine-blood tube to determine intra-assay variability (repeatability). All loading and cell-counting for this portion of the study was performed by a single observer (JW).

#### Inter-assay Variability of the Avian Leukopet^™^

For each blood sample included in the intra-assay variability portion of the study, a second phloxine-blood mixture was mixed, loaded, and read to determine inter-assay variability (reproducibility). These samples were prepared and counted by multiple different individuals to capture inherent inter-observer variability. Only one hemacytometer was read for each blood sample by one individual from the group of different individuals.

### Statistical Analysis

Statistical analysis was conducted in the following manner to test study hypotheses: (1) Assess agreement between hematology parameters determined by EST and LO methods; (2) Determine the analytical variability of the LO method; and (3) Investigate whether biological drivers of leukocyte counts differ between quantification methods. All statistical assessments were performed using commercial software at an alpha value of 0.05 (14) (MedCalc version 18.9, MedCalc software bvba, Ostend, Belgium; R version 3.5.1).

#### Objective 1. Agreement Between Avian Leukopet^™^ and WBC Estimates

Agreement in hematological counts from the LO and EST methods was evaluated using Passing-Bablok regression, Bland-Altman plots, and paired Wilcoxon-Signed Rank tests. Passing-Bablok analysis constructs a linear regression model between the results of two diagnostic methods performed on paired data. Ninety-five percent confidence intervals (95% CI) are produced for the slope and the y-intercept of this model. If the 95% CI for the slope contains one and the 95% CI for the y-intercept contains zero, the diagnostic methods agree. If the CI for the slope does not contain one, proportional error is present and if the CI for the y-intercept does not contain zero, constant error is present. Passing-Bablok is robust to outliers, allows for measurement errors, and makes no distributional assumptions, however, it does assume a high positive correlation between the diagnostic methods (15, 16). To test this assumption, Kendall's tau was determined for each set of test results.

Bland-Altman figures plot the differences between paired results produced by different diagnostic methods against the mean of those results. Limits of agreement (LOA), defined as the mean difference ±1.96 times the standard deviation of the differences, are also plotted. Values above or below the LOA demonstrate poor agreement, and patterns in the data can indicate proportional and systematic error.

#### Objective 2. Avian Leukopet Analytical Variability

Intra-assay variability (repeatability) was determined by calculating the coefficient of variation (CV) for three replicates performed by a single observer. Inter-assay variability (reproducibility) was determined by calculating the CV of those same three replicates read by a single observer and one replicate from a different phloxine-blood mixture made using blood from the same turtle read by an individual from a rotating group of observers. The values used to calculate this inter-assay variability were the average of the first three replicates and the single value produced by the second phloxine-blood mixture. The analytical coefficient of variation (CV_A_) was calculated based on the difference between hemacytometer readings within the same individuals, and the between-turtle coefficient of variation (CV_G_) was calculated as the variation between different blood samples. Generalized linear models were used to determine whether inter or intra-assay variability was associated with turtle health status.

#### Objective 3. Biological Predictors of WBC Data Produced by Different Methods

Continuous variables (hematological values) were assessed for normality using skewness, kurtosis, Q-Q plots, and the Shapiro–Wilk test. Descriptive statistics (mean, standard deviation, and range for normally distributed variables, median, 10th and 90th percentiles for non-normally distributed variables) were tabulated. General linear models were constructed separately for each WBC quantification method using the glm function in R (17). Hematological values were the dependent variables, and independent variables included demographic factors (sex, age class), spatiotemporal factors (month, location), and health classification (apparently healthy vs. unhealthy). Akaike information criterion (AIC) model rankings, used to determine relative quality of statistical models, were then performed to determine the most parsimonious model for each hematological value and leukocyte quantification method using AICmodavg (18).

## Results

### Sample Population

A total of 120 EBT including 46 females, 56 males, and 18 turtles of unknown sex were sampled in May, June July, and August, 2018 at Collison (*n* = 26), Forest Glen (*n* = 17), Kennekuk (*n* = 24), Kickapoo (*n* = 17), and Forbes (*n* = 27). One hundred turtles were classified as adults, 20 were juveniles. Eighteen turtles were classified as unhealthy due to the presence of ocular discharge (*n* = 1), nasal discharge (*n* = 2), aural abscesses (*n* = 2), nodular or erosive lesions on the carapace or hard palate (*n* = 5), fresh injuries (*n* = 4), limb swelling (*n* = 2), and oral plaques (*n* = 2).

### Agreement Analysis

**Figure 1:**
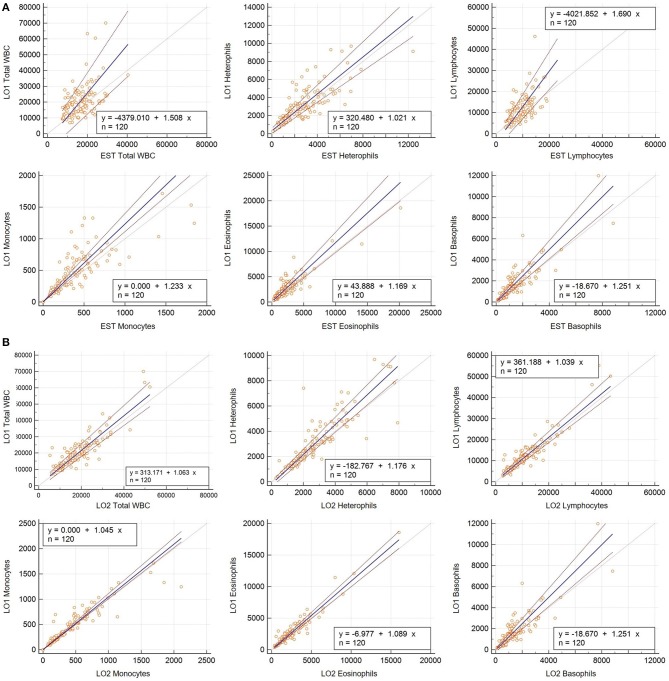
**(A)** Passing-Bablok regression analyses for hematology parameters determined using the Avian Leukopet method with the average of three replicates performed by a single observer (LO1) and the WBC estimation method (EST) in 120 free-living eastern box turtles (*Terrapene carolina carolina*). Solid lines depict linear regression lines and dashed lines depict the 95% confidence intervals around the linear regression line. **(B)** Passing-Bablok regression analyses for hematology parameters determined using the Avian Leukopet method from one observer (LO1) and a group of observers (LO2) in 120 free-living eastern box turtles (*Terrapene carolina carolina*). Solid lines depict linear regression lines and dashed lines depict the 95% confidence intervals around the linear regression line.

**Figure 2:**
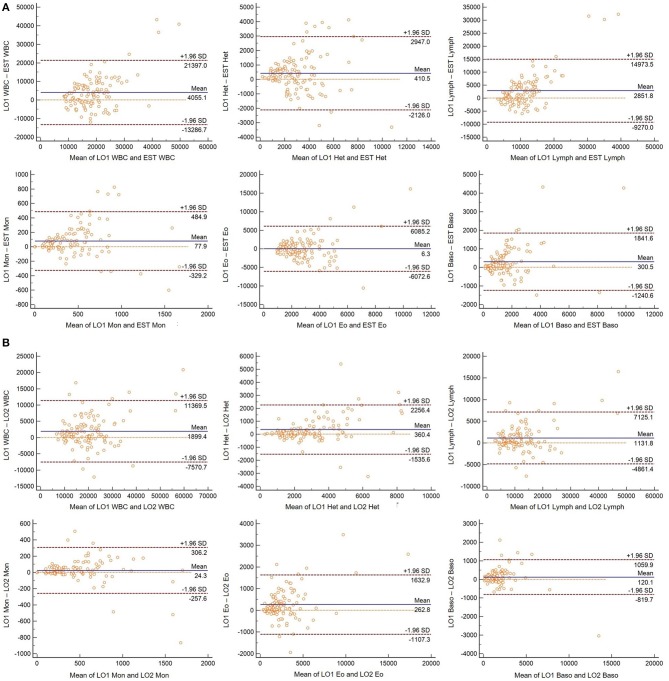
**(A)** Bland-Altman plots for hematology parameters determined using the Avian Leukopet method from one observer (LO1) and the WBC estimation method (EST) in 120 free-living eastern box turtles (*Terrapene carolina carolina*). Central lines depict the mean difference between the two methods, upper and lower lines represent limits of agreement (LOA), defined as the mean difference ±1.96 times the standard deviation of the differences. **(B)** Bland-Altman plots for hematology parameters determined using the Avian Leukopet method from one observer (LO1) and a group of observers (LO2) in 120 free-living eastern box turtles (*Terrapene carolina carolina*). Central lines depict the mean difference between the two methods, upper and lower lines represent limits of agreement (LOA), defined as the mean difference ±1.96 times the standard deviation of the differences.

The LO method (performed by a single observer) resulted in consistently higher absolute leukocyte numbers than the EST method (Table 1, Figures 1A, 2A). Passing-Bablok regression revealed constant and/or proportional error between the LO and EST methods for all leukocytes except eosinophils (Table 2A, Figure 1A). Data for WBC, monocytes, and basophils were proportionally higher in the LO method, heterophils were constantly higher in the LO method, and lymphocytes were both constantly and proportionally increased in the LO method when compared to the EST method.

**Table 1:** Descriptive statistics for leukogram data determined by three different manual count methodologies: estimated leukocyte count from blood films (WBC Estimate), Avian Leukopet^™^ using an average of three replicates performed by a single observer (LO1), and Avian Leukopet^™^ using a single replicate from multiple observers (LO2) for 120 free-living eastern box turtles (*Terrapene carolina carolina*).

		**WBC estimate**	**Avian leukopet^™^ (LO1)**	**Avian leukopet^™^ (LO2)**
**Parameter**	**N**	**Measure of central tendency and dispersion**	**Measure of central tendency and dispersion**	**Measure of central tendency and dispersion**
White blood cells (/μL)	120	16,000 (8,800–24,200)	19,236 (10,321–32,087)	17,129 (9,888–28,800)
Heterophils (H) (/μL)	120	2,553 (847–5,262)	2,811 (1,233–5,709)	2,557 (1,214–4,747)
Lymphocytes (L) (/μL)	120	9,110 (5,176–14,450)	10,844 (5,679–20,364)	10,031 (4,743–19,551)
Monocytes (/μL)	120	290 (0–674)	355 (0–867)	304 (0–850)
Eosinophils (/μL)	120	1,832 (796–4,260)	2,194 (970–4,954)	2,005 (848–4,455)
Basophils (/μL)	120	1,387 (1,156–1,618)	1,688 (1,408–1,967)	1,192 (367–2568)
H:L ratio	120	0.32 (0.28–0.37)		

*Mean and 95% confidence intervals (CI) are presented for normally-distributed parameters, while median and 10th−90th percentiles are presented for non-normally distributed parameters. LO1 values were significantly higher than WBC estimates for each parameter (P < 0.005)*.

**Table 2A:** Passing-Bablok regression parameters and Kendall's Tau values for leukogram data determined using the Avian Leukopet^™^ method (average of three replicates performed by a single observer) and white blood cell estimate from blood films in 120 free-living eastern box turtles (*Terrapene carolina carolina*).

**Parameter**	**Kendall's Tau (*P*-value)**	**Slope (95% CI)**	**Y-Intercept (95% CI)**	**Error present**
White blood cells (/μL)	0.377 (*p* < 0.0001)	1.51 (1.18–1.91)	−4,379 (−11,363, 315)	P
Heterophils (/μL)	0.769 (*p* < 0.0001)	1.02 (0.87–1.20)	320 (4, 598)	C
Lymphocytes (/μL)	0.736 (*p* < 0.0001)	1.69 (1.40–2.03)	−4,021 (−6,992, −1,693)	C, P
Monocytes (/μL)	0.742 (*p* <0.0001)	1.23 (1.12–1.39)	0 (0, 5)	P
Eosinophils (/μL)	0.773 (*p* < 0.0001)	1.17 (1.00–1.35)	44 (−277, 331)	None
Basophils (/μL)	0.837 (*p* < 0.0001)	1.25 (1.07–1.44)	−19 (−198, 88)	P

*C, constant error; P, proportional error*.

There was agreement between LO data determined by a single individual and the group of individuals for total WBC, lymphocytes, and basophils (Table 2B, Figure 1B). The single observer produced proportionally higher values for heterophils, eosinophils, and monocytes. Although, the confidence intervals for these values were narrower than those comparing the LO and EST methods and the observed differences may not equate to significant biological variability (Figure 2B).

**Table 2B:** Passing-Bablok regression parameters and Kendall's Tau values for leukocyte counts determined using the Avian Leukopet^™^ (single observer vs. group of observers) in 120 free-living eastern box turtles (*Terrapene carolina carolina*).

**Parameter**	**Kendall's Tau (P-value)**	**Slope (95% CI)**	**Y-Intercept (95% CI)**	**Error present**
WBC (/μL)	0.671 (*p* < 0.0001)	1.06 (0.95–1.18)	313 (−1345, 1814)	None
Heterophils (/μL)	0.624 (*p* < 0.0001)	1.18 (1.08–1.27)	−183 (−410, 19)	P
Lymphocytes (/μL)	0.493 (*p* < 0.0001)	1.04 (0.95–1.14)	361 (−431, 1039)	None
Monocytes (/μL)	0.856 (*p* < 0.0001)	1.04 (1.01–1.11)	0 (0, 6)	P
Eosinophils (/μL)	0.613 (*p* < 0.0001)	1.09 (1.01–1.17)	−7 (−117, 107)	P
Basophils (/μL)	0.673 (*p* < 0.0001)	1.06 (1.00–1.14)	14 (−38, 68)	None

*C, constant error; P, proportional error*.

### Avian Leukopet^™^ Analytical Variability

Intra-assay variability was 8.2%, while inter-assay variability was 12% (Table 3). Health classification did not significantly impact inter (*p* = 0.99) or intra-assay (*p* = 0.97) CV values.

**Table 3:** Analytical variability of Avian Leukopet^™^ in eastern box turtles (*Terrapene carolina carolina*).

	**Intra-assay variability**	**Inter-assay variability**
N	120	120
Analyzed in triplicate	Yes	No
Mean	20,630	20,155
SD	10,233	9,662
Min	7,016	6,861
Max	70,044	64,826
CV_G_	49.6%	47.9%
CV_A_	8.2%	12.0%
Minimum	0.7%	1.3%
Maximum	21.5%	44.8%

*N, number of samples; Mean, average of total WBC; CV_G_, between-turtle coefficient of variation; CV_A_, analytical coefficient of variation*.

### Biological Effects on Hematological Values Produced Using Different Methods

There was no single general linear model predicting WBC that had overwhelming support (AICcwt > 0.5) for any of the determination methods. In the univariate analysis, age was the only predictor significantly associated with WBC using all three determination methods; juvenile turtles consistently had higher WBC than adults (Tables 4, 5). Location and sex were significant predictors of WBC from the single observer using the LO method but were not statistically significant predictors of WBC using the EST method or the multi-observer LO method.

**Table 4:** Statistical significance of demographic and spatiotemporal factors predicting total white blood cell counts (WBC) in 120 eastern box turtles (*Terrapene carolina carolina*) using three methods for determining WBC.

	**LO1**	**LO2**	**Est**
Month	*P* = 0.499	*P* = 0.34	*P* = 0.46
Location	***P*** **=** **0.03**	*P* = 0.279	*P* = 0.069
Sex	***P*** **=** **0.011**	*P* = 0.0837	*P* = 0.26
Age class	***P*** **=** **0.0002**	***P*** **=** **0.0014**	***P*** **=** **0.00123**
Disease	*P* = 0.51	*P* = 0.81	*P* = 0.685

*P-values are from ANOVAs for month and location and independent t-tests for sex and age class. LO1 = Avian Leukopet method determined in triplicate by a single observer; LO2 = Avian Leukopet method determined by a group of individuals a single time; Est = estimate method to determine total WBC. P-values less than the alpha level (0.05) are bolded*.

**Table 5:** Descriptive statistics for total leukocyte counts which differed by age class in 120 free-living eastern box turtles (*Terrapene carolina carolina*).

		***N***	**Mean**	**Std. deviation**	**95% confidence interval for mean**		**Minimum**	**Maximum**
					**Lower bound**	**Upper bound**		
LO1	Adult	100	19,248	9,390	17,385	21,111	7,017	70,044
	Juvenile	20	27,541	11,669	22,080	33,003	13,830	63,267
	Total	120	20,630	10,234	18,780	22,480	7,017	70,044
LO2	Adult	100	17,620	8,147	16,004	19,237	5,312	52,218
	Juvenile	20	24,282	9,818	19,687	28,877	12,133	49,800
	Total	120	18,731	8,765	17,146	20,315	5,312	52,218
Est	Adult	100	15,848	6,150	14,628	17,068	7,400	40,400
	Juvenile	20	20,210	4,351	18,174	22,246	10,200	28,800
	Total	120	16,575	6,095	15,473	17,677	7,400	40,400

*Mean, standard deviation, 95% confidence interval, and minimum/maximum are presented. LO1 = Total leukocyte count determined using the Avian Leukopet^™^ method determined in triplicate by a single observer; LO2 = Total leukocyte count determined using the Avian Leukopet^™^ method determined by a group of individuals; EST = Total leukocyte count determined using the estimate method*.

## Discussion

This study assessed analytical variability in the Avian Leukopet^™^, compared leukocyte values resulting from two commonly-used quantification methods, and investigated whether methodological differences affected conclusions about biological variation and the health of free-living eastern box turtles. The LO method demonstrated an intra-assay variability of 8.2% and an inter-assay variability of 12%, independent of biological variation. The LO method yielded significantly higher leukocyte numbers than the EST method in an observer-dependent fashion. Similarly, associations between biological predictors and WBC counts differed based on method and observer. This highlights the importance of considering methodology when interpreting hematologic data and underscores the inherent variability of manual hematologic methods in reptiles.

Manual leukocyte quantification is technically challenging, and each method has a different set of benefits and limitations. Both the EST and LO methods require a high-quality blood smear with an even cell distribution along with a knowledgeable slide reader to discern between cells of similar appearance (e.g., lymphocytes vs. thrombocytes). The EST method is inexpensive, requires only a drop of blood to make a smear, and allows for the observation of leukocyte abnormalities such as toxic heterophils or intra-cellular inclusions; however, poor quality smears and anticoagulant artifacts may affect the accuracy of this approach. The advantages of the LO method include rapid identification of stained leukocytes that can be performed by an observer without advanced training or knowledge of hematology if a knowledgeable observer performs the accompanying 100-WBC differential count. However, this method can be time-consuming due to the series of 10 min incubations during phloxine staining and hemacytometer loading. Errors can be made at multiple stages including improper mixing of blood into the stain, error in dilution, and improper charging of the hemacytometer. The EST method has been previously recommended to be routinely run in conjunction with the LO method for verification (19, 20). However, a gold-standard hematological method has yet to be identified in reptiles. Until a gold-standard is available, research efforts should focus on quantifying analytical variability in manual methods to better understand their potential impact on clinical decision-making.

Method-based variation in leukocyte quantification has been previously described in other species with nucleated erythrocytes (6–9, 21). All leukocyte quantification methods in the present study identified significantly higher WBC counts in juvenile turtles. The single-observer LO method also detected associations between location, sex, and WBC count; however, the effects of these variables were small compared to the effect of age class and may represent statistical significance without accompanying biological importance. Deem et al. (8) also found that leukogram interpretation varied based on hematologic method; specifically, WBC counts differed between turtle groups using the Eosinophilic Unopette method (foraging < nesting and stranded), but not the EST method. These findings indicate that hematologic method may influence clinical assessment of reptilian patients, potentially affecting our understanding of biology, physiology, and health at the individual and population levels.

Previous studies have also documented higher WBC counts using phloxine-based stains (Eopettes, eosinophilic Unopettes) compared to EST methods in Galápagos tortoises (6), leatherback sea turtles (9), macaws (21), and loggerhead sea turtles (8). The reason for leukocyte overestimation in the LO method is unknown, but it has been reported that the total WBC can be artificially elevated when using the LO method if the heterophil count is low (22). Many reptiles, including eastern box turtles, have been reported to be predominantly lymphocytic (23–31) which may help explain this finding, although a more recent study reported heterophils as the dominant leukocyte in eastern box turtles (32). These discrepancies indicate that comparing hematologic indices between studies with different methodologies should be approached with caution.

The coefficient of variation is used as a measurement of analytical imprecision in method validation studies (33). The present study is the first to report intra (8.2%) and inter-assay (12%) CV for the LO method in reptiles. These values are consistent with the 6.8% intra-assay CV reported by Dein et al. (34) for avian leukocytes using the BD Unopette method, and with four additional avian studies that reported intra-assay CV values of 11–34%. While box turtle CV values are similar to those in birds, it is unclear whether this level of inherent variability is acceptable for medical use. Criteria for determining acceptable analytical precision can be derived from biological variation data (intra-individual variation; CVi), with the maximum allowable imprecision (CVmax) equating to less than half the CVi (5). Based on human CVi data, manual leukocyte quantification methods produce an unacceptable level of imprecision for clinical decision-making in dogs and cats (5). While CVi data are not currently available for box turtle hematologic indices, these values would be useful to further contextualize and interpret the Avian Leukopet CV values determined in the present study.

Coefficients of variation (analytical imprecision) and bias (systematic error) are also used to calculate total error (TE); a value that represents the entire analytical uncertainty in a diagnostic test (35). Total allowable error (TEa) is defined as the amount of medically tolerable variation for a given clinical pathology analyte. Comparing observed total error (TEobs) for a new diagnostic method to TEa provides an objective means of determining whether the diagnostic method's analytical performance is clinically acceptable (TEobs < TEa) (35). While TEa guidelines have been produced for hematologic indices in veterinary species, bias estimates for the Avian Leukopet in box turtles are unavailable. Determining bias involves comparing paired test results between a new diagnostic method and a gold-standard method using commercially-produced quality control material (QCM) that is not yet available for reptiles (36). Alternative methods for determining bias are recommended when QCM and gold-standard diagnostic methods do not exist, however, these were not pursued in the present study (36). While determining bias and calculating TEobs and TEa was outside the scope of this study, future investigation of these values would be useful for evaluating the clinical validity of the Avian Leukopet in box turtles.

Future directions for research include determining CVi and bias for different hematologic methods in box turtles and other reptiles. This information will enable a more objective comparison between methods and help identify the most valid diagnostic option for clinical assessment. Developing a gold-standard hematologic method is another important milestone which will advance clinical pathology interpretation in reptile patients.

Researchers and clinicians must be cognizant of the potential for method-generated variability when comparing results between diagnostic assays and making clinical decisions. As demonstrated in this study, commonly used methods for reptile hematology may produce significantly different results and biological associations. The analytical variability values documented in this study are an important first step toward understanding the inherent variation in reptile hematologic indices, however, a gold standard method for reptile hematology needs to be established to facilitate method comparison. Continued characterization of manual hematologic methods will ultimately improve clinical case assessment, direct research methodology, and lead to better reptile health management.

## Data Availability Statement

The datasets generated for this study are available on request to the corresponding author.

## Ethics Statement

The animal study was reviewed and approved by University of Illinois Institutional Animal Care and Use Committee.

## Author Contributions

MA and NS: experimental conception and design. MA, LA, JW: sample collection. LA and JW: hematology. MA and LA: statistical analysis. JW, LA, MA, and NS: paper writing.

### Conflict of Interest

The authors declare that the research was conducted in the absence of any commercial or financial relationships that could be construed as a potential conflict of interest.
